# The Proprioceptive Map of the Arm Is Systematic and Stable, but Idiosyncratic

**DOI:** 10.1371/journal.pone.0025214

**Published:** 2011-11-16

**Authors:** Liliana Rincon-Gonzalez, Christopher A. Buneo, Stephen I. Helms Tillery

**Affiliations:** Graduate Program in Biomedical Engineering, School of Biological and Health Systems Engineering, and Department of Psychology, Arizona State University, Tempe, Arizona, United States of America; The University of Western Ontario, Canada

## Abstract

Visual and somatosensory signals participate together in providing an estimate of the hand's spatial location. While the ability of subjects to identify the spatial location of their hand based on visual and proprioceptive signals has previously been characterized, relatively few studies have examined in detail the spatial structure of the proprioceptive map of the arm. Here, we reconstructed and analyzed the spatial structure of the estimation errors that resulted when subjects reported the location of their unseen hand across a 2D horizontal workspace. Hand position estimation was mapped under four conditions: with and without tactile feedback, and with the right and left hands. In the task, we moved each subject's hand to one of 100 targets in the workspace while their eyes were closed. Then, we either a) applied tactile stimulation to the fingertip by allowing the index finger to touch the target or b) as a control, hovered the fingertip 2 cm above the target. After returning the hand to a neutral position, subjects opened their eyes to verbally report where their fingertip had been. We measured and analyzed both the direction and magnitude of the resulting estimation errors. Tactile feedback reduced the magnitude of these estimation errors, but did not change their overall structure. In addition, the spatial structure of these errors was idiosyncratic: each subject had a unique pattern of errors that was stable between hands and over time. Finally, we found that at the population level the magnitude of the estimation errors had a characteristic distribution over the workspace: errors were smallest closer to the body. The stability of estimation errors across conditions and time suggests the brain constructs a proprioceptive map that is reliable, even if it is not necessarily accurate. The idiosyncrasy across subjects emphasizes that each individual constructs a map that is unique to their own experiences.

## Introduction

There is evident value in knowing the spatial location of one's hand, as such knowledge is essential for interacting with our environment. The fact that we position our hand in a spatial context suggests that an external reference frame, fixed to the world, may be important for processing visual and somatosensory signals. The spatial processes that underlie the estimate of hand location appear also to be reflected in movement. For example, the spatial pattern of errors observed with proprioceptive matching is reflected in the pattern of errors in point-to-point movements [1]. Similarly, two groups recently showed a causal link between motor signals and somatosensory systems when motor learning changed the perceived hand position [2], [3]. It remains unclear how visual, proprioceptive, and tactile modalities come together to create the structure of the hand-location map.

Studies that have probed the interactions between these sensory modalities have given us some important insights. Several studies have demonstrated that tactile feedback helps proprioceptive signals in enhancing end-point accuracy and reducing postural sway [4]–[12]. Likewise, postural signals can clearly affect tactile perception [13]–[16]. For example, the spatial interactions between vision and touch have been shown to update with posture of the relevant body part, as long as there is any visual feedback [17]–[22]. Imaging studies have also shown that proprioception plays a role in tuning and updating this visual-tactile map [23], [24].

At the level of single neurons, recordings have also shown interactions between the visual, proprioceptive, and tactile modalities. Visual-tactile neurons discharge with tactile stimuli on the hand and visual stimuli near the same hand, regardless of the position of the hand in space [25]–[28]. More recently, single units in somatosensory cortex have been shown to encode information about both contact with objects as well as movement-related signals [29]. Although it is believed that the body schema used to adjust posture and guide movement relies on both proprioception and vision [30]–[33], estimation of hand location appears to rely on proprioception as the fundamental signal, with tactile and visual signals acting to fine-tune this estimation.

Multiple investigators have examined the ability of subjects to identify the spatial location of their hand based on these signals [1], [4]–[6], [34]–[50]. Despite this, relatively little is known about the resulting spatial structure of the estimation errors. Constructing and analyzing the spatial pattern of error vectors as subjects estimate the location of their hand has proven difficult. In particular, the spatial errors for individual subjects in these tasks are frequently large and so idiosyncratic that it is tempting to draw a conclusion that the analyses have not really captured information about spatial representations *per se* [5]. Instead, one might conclude that the complex patterns of errors observed in previous studies were the result of overfitting noisy data sets. In fact, these noisy errors have even been explicitly discarded as unexplained drift and variability during data analysis in a few cases (see e.g. [40], [51]).

The spatial structure of the estimation errors of individual subjects has not, to our knowledge, been analyzed in detail. Nonetheless, several studies have made casual observations that the estimation errors appear to be remarkably stable, although subject-specific [1], [5], [35], [38], [40], [42], [44], [45], [47]. Despite these repeated observations, analysis of the error patterns in these tasks still tends to focus on generalized effects across subjects. Here we ask whether the patterns truly are subject-specific. If so, this would imply that there is not a single, ideal, proprioceptive map that is acquired by all subjects. Instead, each individual may arrive at a different proprioceptive map based on a unique combination of learning and calibration processes. This would suggest further that many different proprioceptive maps are consistent with accurate and reliable hand position estimation. Consistent with the idea of a calibration of proprioceptive inputs against visual estimates of hand position, other studies have shown that on average, subject estimations are non-uniform across the workspace. That is, errors are smallest when targets are located closer to the body, near the midline, where subjects have the most experience interacting with objects [5], [40], [52]–[55].

We hypothesize here that we estimate the location of our hands in space using an underlying proprioceptive map that is systematic and stable, but subject-specific. In the present study, we report experiments designed to investigate the individual spatial structure of the proprioceptive map. Specifically, we examined the estimation errors across a 2D horizontal workspace that resulted as subjects used visual, proprioceptive, and/or tactile signals to estimate hand location. Performance was tested at 100 target locations across the workspace by having subjects transform solely proprioceptive information about the position of their hands at a target to a solely visual estimate of the same target. We reconstructed and analyzed the individual spatial structure of the resulting estimation errors under four conditions: tactile stimulation, no tactile stimulation, right hand, and left hand. We found that this structure was stable across conditions and time, but unique to each subject.

## Materials and Methods

Seven males and two female subjects between the ages of 20 and 35 participated in two different series of experiments. All subjects were free of upper limb neuromuscular impairment and had normal or corrected-to-normal vision. Six subjects were right handed with the following scores 62.5, 76.5, 78.9, 78.9, 80, and 87.5 in the Edinburgh handedness inventory. Three subjects were left handed with scores of −33.3, −73.3, and −100 according to the Edinburgh handedness inventory. All of the subjects signed written informed consent documents before each experiment. This study was approved by the Institutional Review Board at Arizona State University.

### Experimental setup and procedures

**Figure 1:**
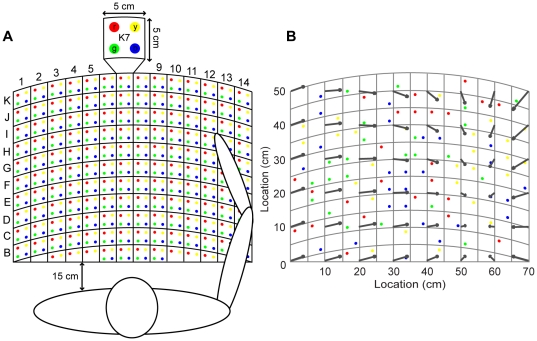
Experimental setup. (A) Each square was labeled with a row letter, a column number, and four colored circles (red, yellow, green, and blue). (B) The colored targets represent an example of a target set. The superimposed vector field represents an example of a spatial structure of mean errors generated with the fourth-order regression. The beginning of the arrow indicates the target where the finger was positioned and the arrowhead indicates where the subject's estimation of the target.

The core task in these experiments was estimation of the 2D location of the index fingertip after it was passively displaced to a target and taken back to the resting position. In order for the subjects to report their estimated hand location without subsequent movement of either arm, we created a 2D grid with labeled locations so that subjects could verbally report fingertip location (Figure 1A). The grid was marked with A through K rows and 1 through 14 columns. Each square on the grid was 5 by 5 cm and had four colored targets, which were 0.4 cm in diameter. There were a total of 616 targets located 1.25 cm apart along the horizontal (x) and depth (y) dimensions.

Subjects sat 15 cm in front of the grid, which was set on a stationary and horizontal table. Each subject was asked to align the body's midline with the grid's midline, which was located between columns 7 and 8. Both hands initially rested on the chair's armrests (resting position), located 2 cm from the edge of the grid. On each trial, the experimenter grasped the subject's wrist, being careful not to touch the hand, and passively moved it to a target where one of two conditions (see below) was applied for about 5 sec. Subjects were asked to keep their eyes closed and their index finger extended during each movement. After the hand was passively brought back to the resting position, the subject was asked to look at the grid and verbally report the grid location where they thought their index finger had been located, without making a reaching movement. Subjects used the column letters, row numbers, and target colors to identify the estimated location (e.g. d5y), and never received feedback regarding the actual location of the target. All of the trials were performed by the same experimenter, who strove to keep the passive displacement constant and without significant change between trials and conditions. No specific path or trajectory was used to move the finger to and from the target. This process was repeated for 100 different targets for each condition and hand. The 100 targets were chosen to be evenly distributed on the grid: an example target set is shown in Figure 1B. The target distribution was varied slightly among subjects to account for differences in arm lengths and depended on which row subjects could reach without moving the torso. There were three different target sets, A, B, C, in which the targets were evenly distributed up to either rows K, J, or I, respectively (see Table 1 for target set assignment). The same target set was used in the same order for the same subject in the Touch and No-Touch conditions and was reflected across the midline for the other hand. Subjects were able to reach any target within the workspace. In all cases, the targets were evenly distributed across the midline.

**Table 1:** Test of Similarity Across Hands and Conditions: Resulting k and p-values from the K-S test.

	Right-Left Hands				No Touch-Touch			
Subject	No Touch		Touch		Right Hand		Left Hand	
(Target set)	k	p	k	p	k	p	k	P
DM (A)	.241**	.009	.326**	1.1E-4	.260**	.004	.405**	4.0E-7
	(.271*)	(.048)	(.542**)	(6.6E-7)	(.229)	(.138)	(.604**)	(1.7E-8)
DH (B)	.305**	7.1E-04	.271**	.003	.432**	2.8E-07	.278**	.002
	(.188)	(.333)	(.27*)	(.04)	(.542**)	(6.6E-7)	(.375**)	(.002)
IK (A)	.329**	1.3E-04	.273**	.003	.385**	1.6E-6	.425**	5.4E-7
	(.583**)	(6.2E-8)	(.542**)	(6.6E-7)	(.458**)	(4.5E-5)	(.521**)	(2.1E-6)
JL (C)	.366**	2.1E-05	.214*	.041	.302**	8.5E-4	.286**	.002
	(.354*)	(.003)	(.271*)	(.048)	(.333**)	(.007)	(.292*)	(.027)
LF (A)	.268**	.003	.207*	.042	.357**	2.8E-05	.293**	6.9E-04
	(.458**)	(4.6E-5)	(.396**)	(7E-4)	(.521**)	(2.1E-6)	(.521**)	(2.1E-6)
MB (C)	.213*	.032	.312**	2.9E-04	.356**	2.0E-05	.319**	2.1E-04
	(2.92*)	(.027)	(.271*)	(.048)	(.438**)	(1.2E-4)	(.333**)	(.007)
NB (A)	.316**	7.5E-05	.361**	4.0E-06	.204*	.029	.423**	3.0E-08
	(.521**)	(2.1E-6)	(.479**)	(1.7E-5)	(.333**)	(.007)	(.729**)	(3.7E-12)
CP (B)	.369**	5.2E-06	.302**	5.0E-04	.289**	8.0E-04	.299**	6.1E-04
	(.583**)	(6.2E-8)	(.375**)	(.002)	(.5**)	(6.1E-6)	(.396**)	(7E-4)
TS (B)	.105	.644	.409**	5.7E-07	.405**	5.2E-07	.163	.157
	(.188)	(.333)	(.604**)	(1.7E-8)	(.5**)	(6.1E-6)	(.208)	(.220)

*p<.05.

**p<.01.

The order of the stimulation conditions was randomly assigned to subjects as they were recruited. The right hand was completed first for all subjects in one block of experiments, and then the same subjects were re-recruited 4 months later to repeat the experiment with their left hand. Each stimulation condition was completed on a separate day.

In the Touch condition the subject received tactile stimulation; the experimenter lightly pressed the subject's fingerpad to a target on the grid and held it there for 5 seconds. In the No-Touch condition the subjects did not receive tactile stimulation. The experimenter held the wrist with the subject's index finger about 2 cm above the target surface for 5 seconds. Although this procedure was not standardized, it was not changed from trial to trial or from experiment to experiment.

### Analysis

**Figure 2:**
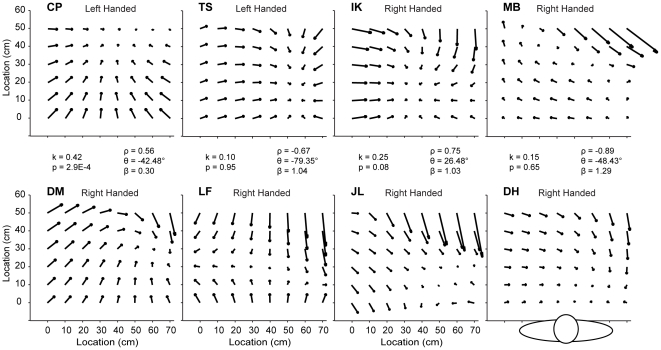
Idiosyncrasy of Pattern of Errors Across Subjects. Distribution of errors from six right-handed and two left-handed subjects when using the Right hand in the Touch condition. Each arrow represents the constant error predicted by the fourth-order regression. The human figure represents the location of a subject with respect to the grid and the resulting pattern of errors. The text in the middle of the figure represents the resulting values from the K-S test and vector correlation analysis for the comparison between the adjacent (above and below) two vector fields.

Performance was evaluated by measuring the direction and magnitude of the errors between the actual and estimated target locations (Figure 2). More specifically, the x and y coordinates of the actual and estimated location of each target were measured and used to calculate error vectors, which in turn were used as estimates of the spatial structure of the proprioceptive map.

We first quantified the degree of similarity between patterns of errors exhibited in different conditions and between subjects. To this end, we used a vector field correlation method for quantifying the effect of subjects, tactile feedback and hand used on the vector field shape and scale [56]. Briefly, this nonparametric method describes the degree of relatedness between two sets of two-dimensional vectors by producing a correlation coefficient, ρ, that is analogous to a scalar correlation coefficient. It also takes into account irregularities and asymmetries in the fields to quantify the degree of rotational or reflectional dependence and the scaling relationship between the vector fields. The correlation coefficient ranges from −1 to 1, which represents a perfect reflectional relationship and a perfect rotational relationship, respectively. This method also provides the angle of rotation that best aligns the vector fields, θ, and a scale factor, β, that describes the scaling relationship between the two fields. Correlating a field with itself would result in a ρ of 1, a θ of 0°, and a β of 1. We used this method to analyze the relationship between two patterns of errors by comparing two vector fields at a time. Note that for comparisons between hands the constant error vector field from one hand was reflected and then superimposed on the error vector field from the other hand. Lastly, as a control analysis, we also performed the correlation after shuffling the vectors in one vector field and pairing them with the vectors in the other field.

**Figure 3:**
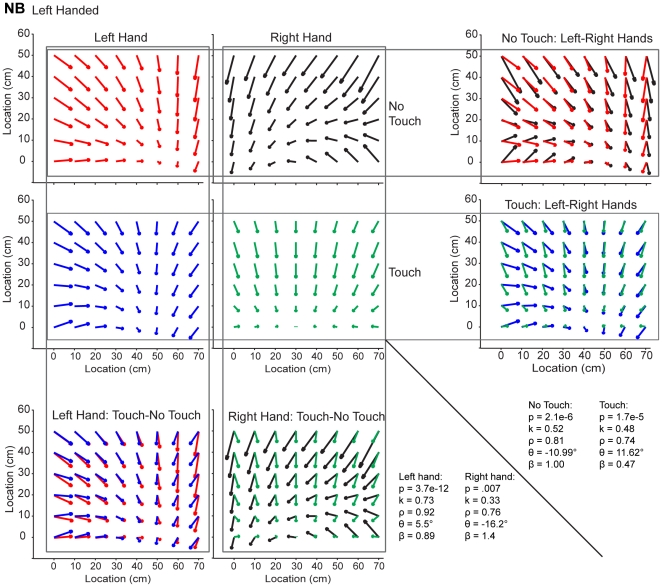
Similarity of Pattern of Errors Across Hands and Conditions. Distribution of errors from one left-handed subject for both hands and both tactile feedback conditions. The text in the right bottom corner represents the resulting values from the K-S test and vector correlation analysis for each of the comparisons in the figure.

The direction of the error vectors was analyzed to determine if the spatial structure of the estimation errors differed significantly between hands, stimulation conditions, and subjects. In order to analyze differences in the spatial structure of the estimation errors between hands, the constant error vector field from one hand was reflected and then superimposed on the error vector field from the other hand for the same condition (see e.g. Figure 3). Then, the absolute angular difference between each of the superimposed vectors was measured. We used the same method, without the reflections, to analyze differences in the spatial structure of the estimation error between stimulation conditions (Touch/No-Touch) for each hand. As a control, the vectors in one of the error vector fields were shuffled and spatially randomized before being superimposed onto the other error vector field. This randomization provided a “null” distribution, which accounted for any overall biases in the pattern of errors for a given subject. The distributions of the two different sets of angles were plotted and analyzed by the Kolmogorov-Smirnov (K-S) test.

**Figure 4:**
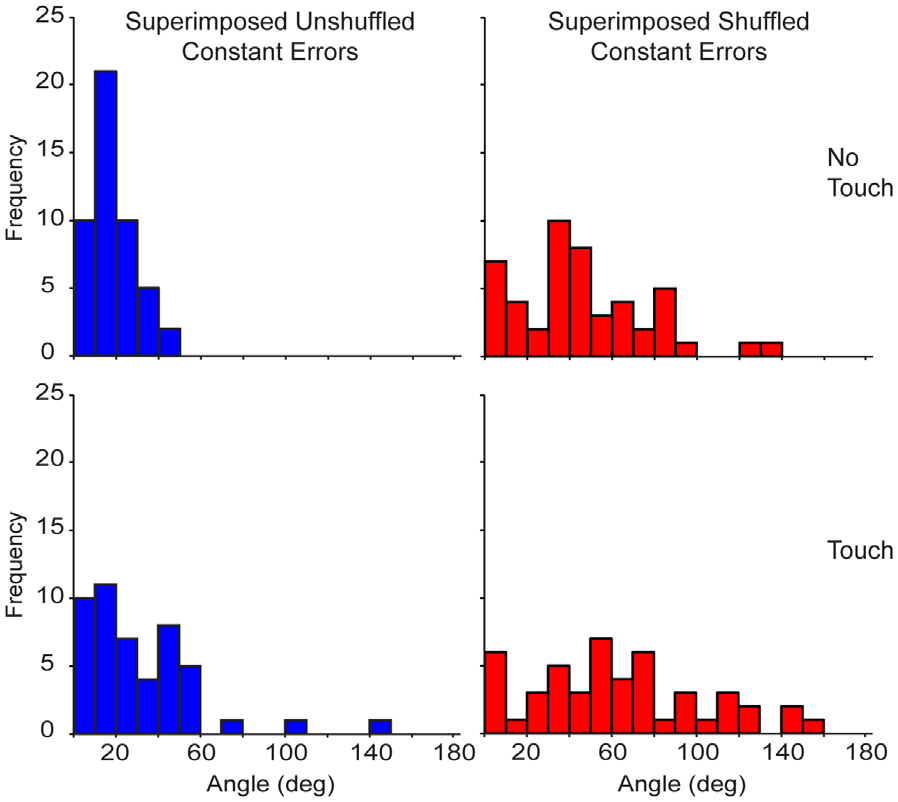
Histograms of the angles between the superimposed vectors. The left histograms show the angle distribution of the superimposed constant error vectors across hands for the subject displayed in Figure 3. The right histograms show the angle distribution of the superimposed vectors when the constant errors from one hand were shuffled before being superimposed.

**Figure 5:**
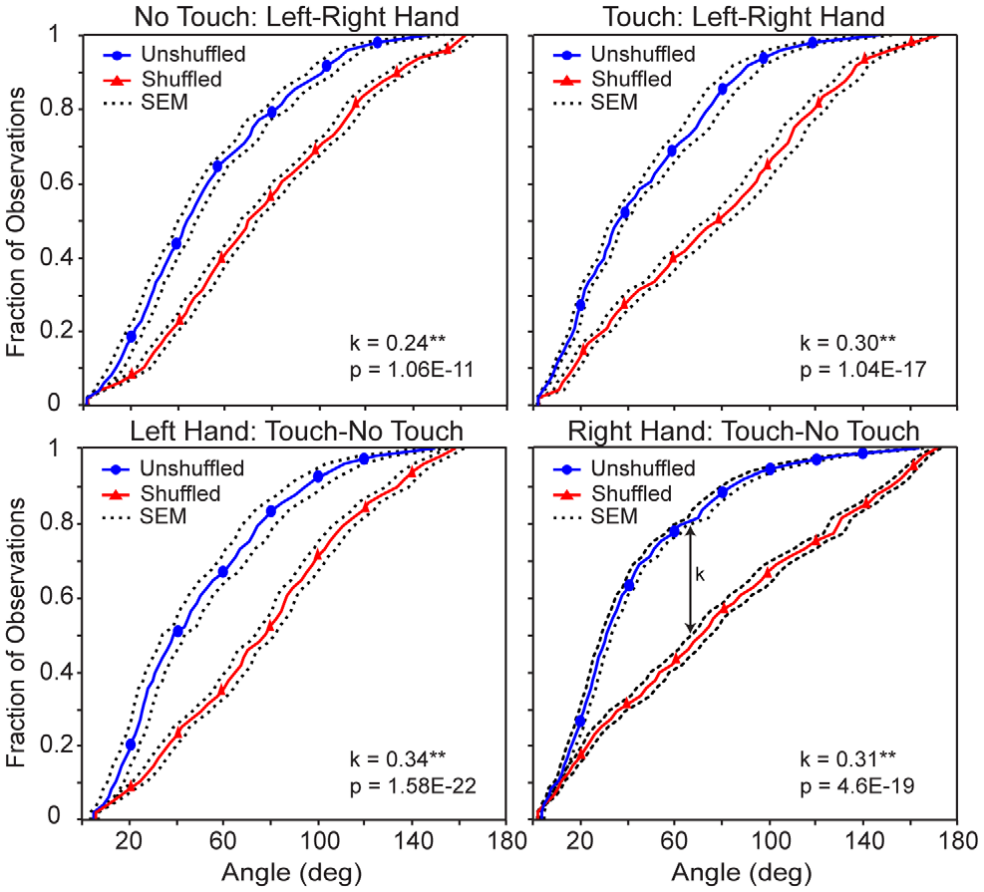
Average Cumulative Distribution of angles. The distributions contain the pooled data from all nine subjects for the angles obtained from the superimposed constant error vectors for both hands and conditions. The k-value represents the greatest distance between the two distributions.

The K-S test measures whether two cumulative distributions are different from each other by finding the greatest difference between the two and assigning it a k-value and a p-value (see e.g. Figure 3). A large k-value and a p-value of less than .05 indicate that the two angle distributions (unshuffled vs. shuffled) are significantly different and that the two vector fields are significantly more similar than would be expected by chance. This provided a measure for the stability of the structure of the estimation errors within-subjects for the four experimental conditions. On the other hand, a non-significant difference in distributions indicates that the two vector fields can be described as no more similar than would be expected by chance (see Figures 4 and 5). This provided a measure for the idiosyncrasy of the performance when comparing the spatial structure of the estimation errors between-subjects. Since there were three different target sets, only those target locations that matched across subjects were used for the K-S test and vector correlation analysis.

In addition to analyzing the direction of the errors, we looked at the accuracy of the performance: we used ANOVA to statistically analyze the magnitudes of the errors. The mixed model had four main factors at different levels and one interaction factor: stimulation (Touch vs. No-Touch), dominance (right-handed vs. left-handed), hand (right hand vs. left hand), subjects (1–9) treated as random variables, and interaction between stimulation and hand. The response in the model consisted of one mean error per factor; each mean error resulted from averaging the 100 errors in each experimental condition. The Tukey's HSD (Honesty Significant Difference) posthoc test was used to test the differences among the least square means (LSmeans) at a significance level of 0.05. JMP software (SAS, Cary, NC, USA) was used to run the model.

Finally, to investigate how the accuracy of performance varied across the workspace, we measured the magnitude of the errors at six different segments in the grid. Lateral location of the targets: left hemifield (x = 0–25 cm), middle (x = 25–45 cm), right hemifield (x = 45–70 cm), and distance from body: near field (y = 0–25 cm), and far field (y = 25–50 cm). This measure was similar to the configuration adopted by Wilson et al. (2010), where proprioceptive bias and acuity was tested at 9 positions for both hands: near, middle, far, left, center, and right [53]. In contrast with their design, subjects in the current study performed the experiment with both hands so it seemed appropriate to test the effect of ipsilateral and contralateral fields. Specifically, we wanted to examine how subjects' accuracy varied between targets that were located closer and farther away from the body and if there was an effect of crossing the midline. As for the analysis described above, we built an ANOVA model to examine these effects. The response in the model consisted of six mean errors per effect; each mean error resulted from averaging all the errors in each of the six segments. The mixed model had six main effects and two interactions. The main effects were: stimulation (Touch vs. No-Touch), dominance (right-handed vs. left-handed), hand (right hand vs. left hand), subjects (1–9) treated as a random variable, lateral location (ipsilateral: right hand in right hemifield and left hand in left hemifield, middle, and contralateral: right hand in left hemifield and left hand in right hemifield), distance from body (near vs. far fields), and interaction between stimulation and hand and also between lateral location and distance from body.

A stepwise regression was used on a 4th order polynomial to build a model of the raw data, which allowed us to estimate consistent errors made by the subjects and to smooth the data for visualization purposes. These errors are referred as ‘constant errors’ throughout the manuscript. Equations were created for each experiment and only contained those parameters that contributed significantly to the fit. This method allowed us to capture spatial regularities in each subject's performance without requiring repeated measures. The model was used to plot the spatial organization of the error vectors by using 48 locations evenly distributed over the target space and contained entirely within the sampled workspace (Figures 1B, 2 and 3). All statistical analyses were performed on both the errors calculated from the raw data and the constant errors obtained from the 4th order regression.

## Results

To investigate the structure of the proprioceptive map used to estimate hand location, subjects were tested across a 2D horizontal grid at 100 target locations. The resulting spatial pattern of estimation errors was analyzed for the right and left hands in the No-Touch and Touch conditions.

### Spatial Structure

Figure 2 shows the constant errors made by six right-handed and two left-handed subjects for the right hand with tactile feedback. Each of the eight panels represents a complete grid with the midline at 35 cm. Subjects aligned themselves with this midline as shown in the bottom right panel. Each constant error is represented with an arrow indicating magnitude and direction. The beginning of the arrow indicates the target where the finger was positioned by the experimenter, and the arrowhead indicates the subject's estimation of that finger position, as predicted by the fourth-order regression. Note the differences between subjects. Each subject appeared to exhibit a spatial pattern of errors that was distinct from that of the other subjects'. For example, all subjects appeared to have points of minimum error that were located in a different workspace location.

Although the patterns of errors across subjects appeared idiosyncratic, there was a striking similarity between hands and Touch/No-Touch conditions for each subject. Figure 3 shows the constant errors made by one left-handed subject at each target location for both hands and tactile stimulation conditions. Note the similarities between the Touch and No-Touch conditions and the near mirror-image symmetry between hands. This subject tended to undershoot faraway targets, resulting in a spatial pattern of errors that points towards the body and contralateral arm.

We used the vector field correlation method to quantify the similarity between hands and conditions. Table 2 shows the mean and standard deviation of the unsigned values of ρ, θ, and β, for each comparison. For θ, circular statistics were used to obtain these values [57]. First, we compared the Touch and No-Touch vector fields for both the right and left hands. The correlation coefficients obtained in most of the individual comparisons were positive, indicating a rotational rather than a reflectional relationship generally existed between the fields. More importantly the mean correlation coefficients and the angles of reflection/rotation showed that tactile feedback did not change the overall structure. That is, on average the vector fields in the two stimulation conditions were highly correlated (ρ = 0.82) with a small angle (θ = 22.00) and a scaling factor close to 1 (β = 0.91). This was especially true when compared to the correlation coefficient, angle, and scaling factor obtained when the vectors in each field were shuffled (Table 2). Interestingly, the vector fields were more highly correlated between stimulation conditions for the right hand (ρ = 0.86, θ = 20.67, β = 1.04) than for the left hand (ρ = 0.79, θ = 23.46, β = 0.77).

**Table 2:** Test of Similarity Across Hands, Conditions, and Subjects: Resulting ρ, θ, β from the vector field correlation analysis of the raw and constant errors.

		Constant Errors				Raw Errors			
		Between Hands	Between Tactile Conditions	Across Subjects	Control: Shuffled	Between Hands	Between Tactile Conditions	Across Subjects	Control: Shuffled
Mean	ρ	0.69	0.82	0.64	0.14	0.37	0.44	0.31	0.10
	θ	22.92	22.00	38.7	69.20	12.97	13.83	30.8	66.90
	β	0.83	0.91	0.70	0.17	0.39	0.48	0.32	0.11
Standard deviation	ρ	0.17	0.13	0.17	0.06	0.1	0.12	0.10	0.04
	θ	21.25	30.53	27.9	40.23	18.48	11.34	26.4	47.60
	β	0.27	0.34	0.34	0.12	0.13	0.15	0.12	0.04

Next we compared the error patterns between the hands within a given stimulation condition. Here again, the individual comparisons generally resulted in positive correlation coefficients. On average, we found that the vector fields were quite similar for this comparison (ρ = 0.69, θ = 22.92, β = 0.83). Note that prior to correlating the fields between hands we first reflected the error vector field from one hand and superimposed it on the error vector field from the other hand. Thus, the relatively high degree of similarity between the fields suggests an approximately mirror image relationship existed between the vector fields for the two hands.

In order to further examine these effects, we calculated the distribution of the angles between error vectors that resulted from superimposing the error vector field from one condition onto those from the other condition. When comparing between hands, we took the mirror image of the error vector field from the left hand and superimposed it onto the error vector field from the right hand. As a null condition, we also measured the distribution of angles resulting when the error vectors from one vector field were shuffled and randomly paired to the error vectors of the other vector field (see Methods). This took into account the fact that the general distribution of errors for many subjects was nonuniform (e.g. subject CP in Figure 2 had a distribution of errors all pointing away from the subject, thus the distribution of angles between two separate conditions could be very nonuniform based merely on that bias). Our null hypothesis was that the two angle distributions (unshuffled vs. shuffled) came from the same distribution, and the alternative hypothesis was that the two angle distributions were from different distributions. Therefore, rejecting the null hypothesis meant that the two vector fields were significantly more similar than would be expected by chance.

Figure 4 shows representative histograms of the angles formed between the superimposed error vectors from both hands for the subject shown in Figure 3. The top histograms correspond to the No-Touch condition and the bottom histograms correspond to the Touch condition. The panels on the left show the angle distribution of the superimposed error vectors from both hands. The panels on the right show the angle distribution of the superimposed vectors when the error vectors from one hand were shuffled before being superimposed. This subject had a higher frequency of smaller angles formed by the unshuffled vectors, indicating that the distribution of errors for both hands was very similar between hands for both conditions. In contrast, the angle distributions created by the shuffled vectors have smaller peaks and look more spread than the histograms on the left. Therefore, the spatial structure of estimation errors created by one hand was similar to the spatial structure of estimation errors created by the other hand. In addition, the same effect was observed when measuring the similarity of the error distributions between stimulation conditions (data not shown).

To verify this effect, we compared the angle distributions using a Kolmogorov-Smirnov (K-S) test. Figure 5 shows the average cumulative distribution of the angles from all nine subjects obtained from the superimposed error vectors. The top two panels show the unshuffled and shuffled distributions that resulted from comparing the vector fields between hands for the No-Touch and Touch conditions. Similarly, the bottom two panels show the distributions that resulted from overlaying and comparing the vector fields across conditions for the Left and Right hands. The k-value represents the greatest distance between the two distributions and is used for the K-S test, which measures whether two distributions are significantly different from each other. The top trace (blue circles) in each panel represents the cumulative distribution of the unshuffled error vectors and the bottom trace (red triangles) represents the cumulative distribution of the shuffled error vectors.

The average distribution of the unshuffled error vectors shows a higher frequency of smaller angles than the distribution of the shuffled error vectors since the cumulative distribution of the former rises faster than the cumulative distribution of the latter. Table 1 shows the results of the K-S test on the raw data and constant errors (between parentheses) from the regressions for each subject when comparing the spatial structure of the estimation errors between hands and conditions. The resulting angle distributions from the unshuffled and shuffled constant and raw error vector fields between hands were significantly different in most instances. Specifically, the spatial structure of constant estimation errors of all but 4 comparisons were significantly more similar between hands and conditions than would be expected by chance. Similarly, the spatial structure of raw estimation errors of all but 2 comparisons were significantly more similar between hands and conditions than would be expected by chance. In addition, the spatial structure had a significant degree of similarity between hands, which suggests an approximately mirror image relationship existed between the vector fields for the two hands. Since these measures were separated by four months, this also tells us that the structure was stable across time.

In addition to measuring the similarity between hands and stimulation conditions, we also quantified the idiosyncrasy of the spatial structure of the estimation errors. This was done by comparing the distribution of angles formed when the error vector field for one hand and one condition from one subject was superimposed onto the error vector field for the same hand and condition from another subject. Only the error vector fields for one condition and one hand were paired at a time and each subject was compared to every other subject separately, resulting in 144 comparisons. As explained above, failure to reject the null hypothesis meant that the vector fields from the two subjects being compared were no more similar than would be expected by chance, and were thus idiosyncratic. Tables 3 and 4 show the results of the K-S test when comparing the spatial structure of the estimation (raw and constant) errors between subjects for all conditions, and for left (Table 3) and right (Table 4) hands. Table 3 shows the results of the K-S test when subjects used the Left hand. The p-values above the diagonal come from the K-S test between subjects for the Left hand and Touch condition, while the p-values below the diagonal come from the K-S test between subjects for the Left hand and No-Touch condition. Similarly, Table 4 shows the two sets of p-values for each pair of subjects compared when they used the Right hand with and without tactile feedback. Out of the 144 comparisons, only 3 (2%) comparisons exhibited a non-idiosyncratic distribution of raw errors, and only 14 (9.7%) comparisons exhibited a non-idiosyncratic distribution of constant errors. The overall spatial structure of the estimation errors was significantly no more similar than would be expected by chance. In other words, the spatial structure of subjects'estimation errors was idiosyncratic.

**Table 3:** Test of Similarity Between Subjects for the Left hand: Resulting p-values from the K-S test.

Left Hand	Touch									
No Touch	Subjects	DM	DH	IK	JL	LF	MB	NB	CP	TS
	DM		.95	.08	.30	.14	.47	.08	.21	.24
			(.82)	(.22)	(.33)	(.48)	(.14)	(.33)	(.14)	(4.6E-5**)
	DH	.42		.84	.52	.29	.08	.59	.51	.26
		(.95)		(.82)	(.65)	(.14)	(.82)	(.48)	(.82)	(.14)
	IK	.05	.13		.06	.19	.38	.14	.11	.08
		(.08)	(.33)		(.33)	(.95)	(.95)	(.22)	(.33)	(.08)
	JL	.21	.51	.22		.45	.64	.07	.07	.27
		(.95)	(.33)	(.14)		(.33)	(.65)	(.08)	(.22)	(.22)
	LF	.70	.97	.78	.06		.08	.08	.48	.07
		(.14)	(.95)	(4.6E-5**)	(.33)		(.08)	(.14)	(.14)	(.48)
	MB	.07	.05	.27	.35	.23		.76	.06	.99
		(2.9E-4**)	(.65)	(2.9E-4**)	(.22)	(.08)		(.22)	(.08)	(.33)
	NB	.07	1.00	.16	.45	.70	.37		.16	.49
		(.22)	(.82)	(.08)	(.33)	(.14)	(.22)		(.08)	(.08)
	CP	.43	.75	.08	.08	.39	.16	.07		.35
		(.08)	(.82)	(4.6E-5**)	(6.6E-7**)	(1.2E-4**)	(3.4E-3**)	(.08)		(.14)
	TS	.92	.46	.64	.46	.28	.49	.86	.75	
		(.82)	(.48)	(.82)	(.99)	(1)	(.82)	(.65)	(.82)	

**Table 4:** Test of Similarity Between Subjects for the Right hand: Resulting p-values from the K-S test.

Right Hand	Touch									
No Touch	Subjects	DM	DH	IK	JL	LF	MB	NB	CP	TS
	DM		.09	.12	.12	1.00	.06	.21	.17	.06
			(.08)	(.08)	(.22)	(.14)	(.14)	(.22)	(2.9E-4**)	(.22)
	DH	.34		3.7E-4**	.35	.99	.10	.19	5.1E-3**	.08
		(.33)		(.14)	(.22)	(.48)	(.65)	(7E-3**)	(.08)	(1.2E-4**)
	IK	.06	.06		.57	.99	.72	.20	.23	.05
		(.33)	(.83)		(.08)	(.14)	(.33)	(6.9E-4**)	(6.9E-4**)	(.14)
	JL	.79	6.3E-4**	.27		.72	.18	.76	.51	.54
		(.22)	(3.4E-3**)	(.83)		(.08)	(.65)	(.65)	(.65)	(.33)
	LF	.76	.11	.25	.24		.32	.95	.51	.76
		(.82)	(.08)	(.08)	(.48)		(.33)	(.82)	(.82)	(.95)
	MB	.78	.97	.89	.59	.35		.87	.72	.29
		(.08)	(.48)	(.33)	(.33)	(.48)		(.33)	(.48)	(.22)
	NB	.87	.32	.07	.06	.48	.91		.07	.13
		(.95)	(.08)	(.08)	(.08)	(.14)	(.95)		(.08)	(.08)
	CP	.77	.06	.06	.07	.23	.78	.20		.07
		(.48)	(.83)	(.83)	(.83)	(.08)	(.22)	(.14)		(.08)
	TS	.20	.09	.24	.09	.56	1.00	.22	.36	
		(.22)	(.14)	(.33)	(.14)	(.14)	(.82)	(.48)	(.14)	

*p<.05.

**p<.01.

The vector field correlation analysis also supports this conclusion. Table 2 shows that on average the vector fields were less strongly correlated between subjects than between conditions and hands for the same subject. Similarly, the scaling factor was smaller (farther from 1) between the vector fields of two subjects than within one subject. In general, comparisons across subjects were better correlated for the Right hand and Touch condition than any other condition (Right hand, T: ρ = 0.70, θ = 47.41, β = 0.75; Left hand, T: ρ = 0.65, θ = 31.81, β = 0.69; Right hand, NT: ρ = 0.60, θ = 41.19, β = 0.62; Left hand, NT: ρ = 0.63, θ = 34.93, β = 0.72). In these set of comparisons, we observed 76 negative correlation coefficients for the constant errors and 52 for the raw errors.

### Magnitude of the error

We measured the mean errors made by each subject in order to verify whether the Touch condition had an effect on reducing the magnitude of the errors and thus on accuracy. We also measured the effect of using either hand on improving accuracy. Table 5 shows the results from the fixed factor ANOVA, which resulted in a mean error of 5.49 cm, an R^2^ of 0.73 and an R^2^-adjusted of 0.69. Only the effects of stimulation, and the interaction of stimulation and hand (Stim X H) were significant. The mean error was significantly lower in the Touch (5.21 cm) condition than in the No-Touch (5.78 cm) condition. However, hand used, hand dominance, and interactions with hand dominance had no effects in the model and had no significant interactions with the other factors. On the other hand, the post-hoc test on the stimulation and hand interaction effect revealed that when subjects used their right hand, the tactile condition was statistically more accurate than when using the right hand with no tactile feedback; this difference did not exist for the left hand. (Post-hoc Stats: p<.05, LSmean (T, R) = 5.02*, LSmean (N, R) = 6.14*, LSmean (T, L) = 5.40, LSmean (NT, L) = 5.42, std error = 0.32).

**Table 5:** Analysis of Variance for the accuracy of the average hand estimation.

Source	DF	F Ratio	Prob>F
Stimulation (Stim)	1	6.57*	0.02
Hand (H)	1	0.17	0.69
Dominance (D)	1	0.01	0.96
Stim X H	1	6.10*	0.03

*p<.05.

**p<.01.

Finally, to investigate how accuracy of estimating hand location varied across the workspace, we measured the magnitude of the estimation errors at six different segments in the grid. Specifically, we wanted to examine whether distance from the body or lateral target location on the workspace had an effect on accuracy. Table 6 shows the results of the fixed factor ANOVA on the divided grid, which resulted in an R^2^ of 0.55 and an R^2^-adjusted of 0.53.

**Table 6:** Analysis of Variance for the uniformity of the accuracy across the workspace.

Source	DF	F Ratio	Prob>F
Stimulation (Stim)	1	15.59**	0.0001
Hand (H)	1	0.66	0.42
Dominance (D)	1	1.60	0.24
Lateral Location (LL)	2	5.98**	<.01
Distance from Body (DB)	1	113.46**	<.0001
Stim X H	1	14.52**	<.001
LL X DB	2	4.96**	<.01

*p<.05.

**p<.01.

As observed with the pooled vectors in the workspace, the ANOVA on the divided grid revealed significant effects of stimulation conditions, target location on the grid, and the interaction of stimulation and hand as well as the interaction of lateral location and distance from the body factors (Table 6). Regarding the main effect of stimulation conditions and its interaction with the hand used, we observed the same effect as described above. (Post-hoc Stats: p<.05, LSmean (T, R) = 4.99*, LSmean (N, R) = 6.19*, LSmean (T, L) = 5.45, LSmean (NT, L) = 5.48, std error = 0.28).

We found that the magnitude of the estimation errors was not uniform across the workspace for all subjects. When analyzing the distance from the body effect (Near: all x's and y = 0–25 cm; Far: all x's and y = 25–50 cm), subjects were more accurate at estimating targets that were located closer to their bodies (p<.0001, LSmean (Near) = 4.71, LSmean (Far) = 6.35, std error = 0.26). When analyzing the lateral location of the targets effect (left hemifield: all y's and x = 0–25 cm; middle: all y's and x = 25–45 cm; right hemifield: all y's and x = 45–70 cm), the performance at the middle location was significantly different than at the contralateral location (LSmean (Middle) = 5.23*, LSmean (Ipsi) = 5.48, LSmean (Contra) = 5.88*, std error = 0.27). Subjects were most accurate at estimating hand location at targets located directly in front of their bodies (middle of the grid).

**Figure 6:**
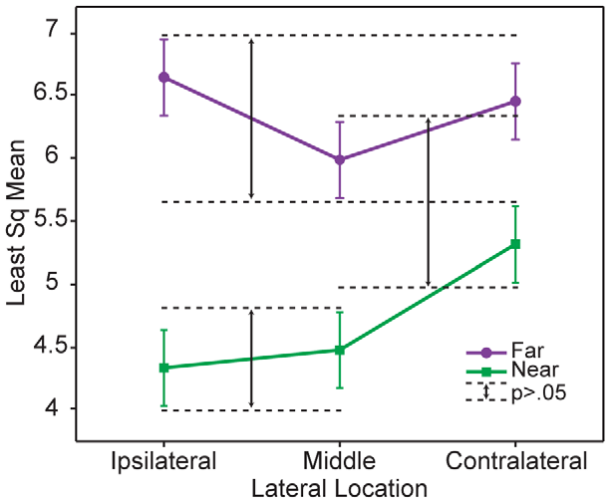
Proprioceptive Accuracy as a function of Lateral Location and Distance from the Body. Analysis of variance of the average error magnitude at 6 different locations on the grid. Lateral location of the targets: Ipsilateral (x = 0–30 cm), Middle (x = 30–40 cm), Contralateral (x = 40–70 cm), and distance from body: Near field (y = 0–25 cm), and Far field (x = 25–50 cm). LSMeans Differences Tukey HSD posthoc test on the significant interaction between lateral location of targets and distance from the body revealed significant interactions between different locations on the grid. Interactions found between the dotted lines are not significant at a p<.05. Everything else is significantly different.

In addition, the interaction between the lateral location of targets and distance from the body (grid divided into 6 segments) was significant (LSmean (Ipsi, Near) = 4.33, LSmean (Middle, Near) = 4.47, LSmean (Contra, Near) = 5.31, LSmean (Middle, Far) = 5.98, LSmean (Contra, Far) = 6.44, LSmean (Ipsi, Far) = 6.63, std error = 0.30). Figure 6 shows the interaction effect in which subjects were more accurate at estimating hand location when the targets were near the body and in the ipsilateral near field. Crossing the midline resulted in significantly less accurate estimations when in the near field. This effect of crossing the midline was not significant when the targets were located farther away from the body.

## Discussion

In this study we investigated the proprioceptive map of arm position information by reconstructing and analyzing the individual spatial structure of endpoint estimation errors under four conditions: with and without tactile feedback, and with the right and left hands. We also examined the dependence of the results on handedness. We found that tactile feedback improved subjects' ability to accurately estimate hand location but did not affect the directional pattern of the errors. While we observed that the effect of tactile feedback was limited to the right hand, handedness had no effect on subjects' accuracy. We also found that the spatial structure of the direction of the errors was stable across conditions and time. Furthermore, we showed statistically that the spatial structure of the estimation errors was idiosyncratic: each subject had a unique spatial structure of estimation errors. Finally, as has been previously shown, we found that the magnitude of the errors had a characteristic and non-uniform distribution over the workspace: errors were smallest close to the body and closer to the body midline. We argue here that these observations are consistent with a proprioceptive map that is constructed by experience using one systematic and stable but idiosyncratic algorithm that is constantly being recalibrated against visual signals.

Although tactile input did not alter the overall structure of the proprioceptive map as seen in the significantly similar fields in the K-S test and highly correlated vector fields, we did find, in agreement with previous studies, that tactile feedback improved the accuracy of hand location estimates [1], [4]–[12]. We found this to be a significant effect whether we looked at the errors across the entire workspace, or when the errors were examined separately for 6 different segments of the workspace. In addition, both ANOVA tests showed that when subjects used their right hand, the tactile condition was statistically more accurate than the no tactile stimulation condition; this difference neither existed for the left hand nor depended on handedness. In agreement with the ANOVA result, the vector fields were shown to be better correlated between subjects in the right hand and Touch condition and within subjects across stimulation conditions for the right hand. These results are contrary to what we expected based on previous studies [48], [49], [58]–[65], which have shown that the nondominant system is better at controlling limb position. On the other hand, Wilson et al. (2010) reported better acuity for the right arm in a proprioceptive matching task [53]. The heterogeneity of these findings in the literature is likely due to the differences in experimental procedures. The studies by Goble and colleagues used proprioceptive target matching tasks, while the studies by Sainburg and colleagues used reaching movement tasks, and the current study used a proprioceptive to visual transformation of target location. In any case, our results do not imply that touch perception is independent from proprioception: touch appears to be body-referenced and moves with the body (e.g. tactile perception depends on hand posture, [16], [66], [67]).

Our key observation is that the spatial structure of the estimation errors is stable across multiple measurements. First, it is symmetric between the hands. That is, the errors made with the right hand looked like an approximate mirror image of the errors made with the left hand, irrespective of the tactile conditions. Here, when we compared the vector field from one hand with a reflected version of the vector field from the other hand we found that the two fields were well correlated. We also showed statistically that the spatial structure of estimation errors was more similar between hands than would be expected by chance. This finding agrees with previous observations that hand biases were mirror-symmetric, which suggested that subjects represent their limbs in space by two separate frames of reference originating at each shoulder [68], [69]. Thus, even though each arm operates in its own egocentric space, it appears that the computations based on the posture of the arms use one algorithm to build the spatial map. The fact that the two arms exhibit mirror-image patterns suggests that this egocentric space is anchored at the shoulder and that this idiosyncratic computation is performed in the same way for each arm. Recent work from Fuentes and Bastian (2010) suggests which variables are important for this computation: proprioceptive biases are dependent on joint configuration and are independent of the task [54]. Finally, the spatial structure of this map is stable not just across tasks, but over time. That is, the spatial structure of the estimation error was not substantially affected when subjects were re-recruited 4 months after the initial set of experiments. Thus, there is one systematic and stable solution to building the proprioceptive map of hand location.

The fact that the spatial structure of the estimation errors was significantly different across subjects suggests that each individual's map is uniquely constructed through a learning mechanism and is thus the result of individual experience. This is in agreement with previous reports: in an endpoint position matching task, dancers showed better integration of proprioceptive signals and also relied more on proprioceptive signals than visual signals compared to non-dancers [69]; in a bimanual parallelity task, what subjects haptically perceived as parallel was influenced by job experience or education [70]. In addition, our results statistically validate casual observations in the literature that the pattern of errors is subject specific [1], [5], [37], [39], [41], [44], [46], [69]. The repeatability of these patterns across conditions and time shows that the patterns are not statistical anomalies resulting from overfitting of noisy data. Rather, the idiosyncrasy is a fundamental byproduct of how proprioceptive information is processed. Both the idiosyncrasy and common features in the spatial structure can be seen in the vector correlation analysis across subjects as the vector fields between subjects were less correlated than the vector fields within subjects, yet, more correlated than the control condition.

While we have focused on the idiosyncrasy, our results do not contradict prior results showing overall patterns in pooled data. In fact, the overall distribution of error magnitudes, as shown when we divided the grid into 6 spaces, is comparable to that shown by Wilson et al. (2010) where proprioceptive bias and acuity was tested at 9 positions for both hands [52]. In agreement with this study and another study by van Beers et al. (1998) [39],we found that all subjects were more accurate at estimating the location of their hands when the targets were closer to the body.

These observations on the structure of the pooled map suggest that the spatial structure of the estimation errors is a consequence of a system that is continually calibrating the proprioceptive map of hand location against the visual representation. The area where we have the most experience interacting with objects (close to the body, near the midline) is where the calibration appears best, and the calibration decreases as you go away from that location. The fact that the idiosyncrasy in the pattern of errors exists for locations close to the body, where the system is highly calibrated across subjects, suggests that the map is based on a general mechanism for estimating hand location given arm configuration: the larger errors at the periphery shape the entire pattern of errors, instead of being limited to the periphery which one might expect in the case of a set of local solutions. Based on these ideas, local perturbations to the structure of the map should propagate throughout the map just like the idiosyncrasy of the errors.

The results presented here provide insight into the structure of the proprioceptive map of the arm: it is systematic and stable, but idiosyncratic. The stability of estimation errors across conditions and time suggests the brain constructs a proprioceptive map that is reliable, even if it is not necessarily accurate. The idiosyncrasy across subjects emphasizes that each individual constructs a map that is unique to their own experiences. Finally, the commonalities seen across subjects suggest that the system is continually being calibrated against other sensory signals.

Taken together, this study highlights the value of studying individual differences in motor performance. Idiosyncrasies might be crucial in allowing us to understand how the central nervous system constructs and uses this map of arm location. Furthermore, this knowledge could be critical in the design of neuroprosthetic devices capable of somatosensory feedback.
